# Scale‐dependent effects of species diversity on aboveground biomass and productivity in a subtropical broadleaved forest on Mt. Huangshan

**DOI:** 10.1002/ece3.9786

**Published:** 2023-02-01

**Authors:** Lei Xie, Hao Chen, Lai Wei, Shuifei Chen, Lu Wang, Baokun Xu, Xiangui Yi, Xianrong Wang, Hui Ding, Yanming Fang

**Affiliations:** ^1^ Co‐Innovation Center for Sustainable Forestry in Southern China, College of Biology and the Environment, Key Laboratory of State Forestry and Grassland Administration on Subtropical Forest Biodiversity Conservation Nanjing Forestry University Nanjing China; ^2^ Research Center for Biodiversity Conservation and Biosafety, State Environmental Protection Scientific Observation and Research Station for Ecological Environment of Wuyi Mountains, Biodiversity Comprehensive Observation Station for Wuyi Mountains, State Environmental Protection Key Laboratory on Biosafety Nanjing Institute of Environmental Sciences, Ministry of Ecology and Environment of China Nanjing China

**Keywords:** abiotic factors, aboveground biomass, biodiversity, biotic factors, ecosystem functioning, forest dynamics, productivity, scale‐dependent effects

## Abstract

The relationship between species diversity and biomass/productivity is a major scientific question in ecology. Exploring this relationship is essential to understanding the mechanisms underpinning the maintenance of biodiversity. Positive, negative, and neutral relationships have been identified in controlled experiments and observational research. However, increasing evidence suggests that the effects of species diversity on aboveground biomass and productivity are influenced by biotic and abiotic factors, but it remains unclear whether scale‐dependent effects affect aboveground biomass and productivity. Herein, we used a generalized linear regression model and a structural equation model to explore relationships between species diversity and productivity/aboveground biomass under different scales and to investigate the effects of topographical factors and species diversity on ecosystem functioning. The results revealed a positive relationship between biodiversity and ecosystem functioning based on species diversity and aboveground biomass. Different sampling scales may impact the relationship between species diversity and ecosystem functioning. A positive relationship was found between species richness and productivity at medium and large scales; however, ambiguous relationships were found in productivity and other species diversity indices. Elevation was a key factor affecting both biomass and productivity. These results suggest that species diversity is not the only factor affecting biomass and productivity, and the positive correlation between species diversity and ecosystem functioning is mediated by abiotic factors.

## Graphical abstract

Different sampling scales may impact the relationship between species diversity and productivity, and elevation was a key factor affecting both biomass and productivity. Species diversity is not the only factor affecting biomass and productivity, and the positive correlation between species diversity and ecosystem functioning is mediated by abiotic factors.

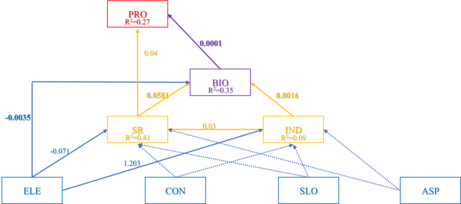

## 1 INTRODUCTION

Forest ecosystems are the most important species resource pools for biodiversity maintenance in terrestrial ecosystems, and they play an important role in regulating the global carbon cycle (Kurz & Apps, 1993; Waring & Running, 2010). It was estimated that the total amount of carbon in aboveground forest ecosystems is 4.83 × 10^14^ kg, accounting for 86% of the carbon content in the global terrestrial ecosystem (Olson et al., 1983). Species diversity, a measurable indicator of a community, reflects the basic characteristics of an ecosystem, and is the result of competition or species coexistence (Gaston, 2000; Oommen & Shanker, 2005). Species diversity provides a material basis and supports conditions for the operation and maintenance of ecosystem functioning. Therefore, exploring the relationship between biodiversity and ecosystem functioning (BEF) is essential to understand the mechanisms underpinning biodiversity maintenance in terrestrial ecosystems.

Surrounding the impact of plant diversity on ecosystem function to be explained by two main hypotheses. The niche complementary hypothesis states that increasing species diversity may well promote niche differentiation for better utilization of resource pools, which would increase the ecological function such as tree carbon storage (Li, Bao, et al., 2019; Tilman, 1997; Tilman et al., 1997). The identity effect states that taxonomy of species with different attributes determines ecosystem functioning, not species richness (Lawton, 1994). Therefore, when species richness is reduced, ecological functioning is affected. In this case, species diversity and ecosystem functioning may be uncorrelated or not monotonic. These two hypotheses are not mutually exclusive and are sometimes complementary. Numerous control experiments excluding the influence of disturbance and environmental factors in nature have been performed to explore the relationship between biomass/productivity and species diversity (Cavanaugh et al., 2014; Poorter et al., 2015). Positive, negative, unimodal, U‐shaped, and neutral relationship hypotheses have been tested (Gillman & Wright, 2006; Mittelbach et al., 2001; Waide et al., 1999). Herbs have a short life cycle and are easy to sample; hence, they are often used to test BEF in grassland ecosystems, and unimodal curves are the most common. In the early stage of community assembly, the differentiation of species in resource utilization leads to a more optimized ecosystem function. As the number of species increases, functions overlap among species, resulting in a decline in ecosystem function. Therefore, species diversity and ecosystem function tend to first increase and then decrease (Poorter et al., 2015).

Conversely, trees have a long‐life history, sampling can cause great damage to forest ecosystems, and allometric equations obtained for different geographical locations can be inconsistent, which poses a great problem when investigating BEF. Therefore, few studies have been conducted on the relationship between species diversity and biomass/productivity in forest ecosystems, especially in forestry dynamics plot in subtropical forest. Allometric growth equations based on 701 woody species determined within 24 large‐scale forest dynamics plots (Forest Global Earth Observatory, ForestGEO) facilitated the estimation of biomass of species in different forest ecosystems, especially subtropical evergreen broadleaved forests (Gonzalez‐Akre et al., 2021). The advantage of this method is that variance caused by sample size, climate, and taxonomy is considered and incorporated into the allometric equation in a weighted manner (Gonzalez‐Akre et al., 2021). On the other hand, we paid too much attention to the effects of species richness on ecosystem function, which alters densities due to interactions between species being overlooked. Therefore, the effects of other alpha diversity indices such as species evenness and the Simpson index on ecosystem function were ignored. For example, the Shannon index represents the evenness of individual distributions among species, and we can infer the interactions of ecological processes and species that affect productivity in the plot based on the relationship between the Shannon index and productivity (Poorter et al., 2015).

However, observational studies showed that biodiversity is not the only driver of biomass/productivity; parallel effects of environmental heterogeneity on biodiversity and ecosystem functioning can lead to changes in BEF. Studies revealed that the relationship between ecosystem functioning and species diversity can be inconsistent at different ecological scales (Chase & Leibold, 2002; Weiher, 1999). A positive relationship is generally observed for small spatial grains, whereas the BEF relationship is ambiguous for large spatial grains (Chisholm et al., 2013). This is mainly because different ecological scales differ in terms of resource competition such as soil fertility and ecological process such as disturbance and complementarity (Chave, 2013; Chisholm et al., 2013). One study predicted that at the regional scale, when species diversity is low, resource complementarity is greater than resource competition (Bond & Chase, 2002). When species diversity is high, resource competition is high, resulting in a unimodal relationship between species diversity and ecosystem functioning (Zhang & Zhang, 2003). At the regional scale, species diversity is positively correlated with ecosystem functioning due to the complementarity of species through migration between different regional patches (Zhang & Zhang, 2003). However, at the local scale, the relationship between species diversity and ecosystem functioning can differ between sampling areas, and the results are unclear. Positive, negative, and no relationship were observed for different small spatial grains (Chisholm et al., 2013). First, the community complexity of the control experiment is far less than that of the natural forest community, especially for subtropical forest, which leads to doubts about whether previous conclusions are applicable to natural forests (Tan et al., 2017). In addition, the importance of different ecological processes in community assembly varies with spatial scales, causing changes in ecosystem function (Chisholm et al., 2013). Therefore, it is necessary to explore the impact of different scale effects on the relationship between species diversity and ecosystem functioning at the local scale. Clearly, different habitats may have an impact on biomass and productivity (Lin et al., 2012).

Few control experimental projects on tree BEF have been reported, and most have focused on tropical, temperate, and boreal biomes (see www.treedivnet.ugent.be). BEF‐China and Competition and Diversity Experiment (CADE) are the only two control experimental projects located in the China. However, no such report has been seen in the subtropical observation platform. Mt. Huangshan Forest, a relatively well‐preserved natural subtropical forest in China, is of great significance for studying biodiversity maintenance and ecosystem functioning (Lv et al., 2022). We previously established a 10.24 ha subtropical evergreen broadleaved forest plot in 2014 based on the Center for Tropical Forest Sciences (CTFS) approach (Ding et al., 2016; Xie et al., 2022). Herein, we used species distribution and DBH data recorded in a forest dynamics plot to explore the effects of different scales on the relationship between species diversity and biomass/productivity at a local scale. We hypothesized that at the local scale, different sampling areas would be characterized by differences in habitat heterogeneity, resulting in relationships between species diversity and ecosystem functioning being scale‐dependent. The relative importance of the two mechanisms, the niche complementarity hypothesis and the identity effect, may also be scale‐dependent. Based on the niche complementarity hypothesis, we predicted that environmental heterogeneity would have an impact on differences between the functioning of species within a community assembly, indirectly affecting biomass and productivity. This study specifically explored (1) whether there are correlations between different levels of species diversity such as species richness and shannon diversity and biomass/productivity, (2) whether there are scale‐dependent effects of species diversity on aboveground biomass and productivity in a subtropical forest at Mt. Huangshan, and (3) whether biotic and abiotic factors affect species diversity and ecosystem functioning.

## 2 MATERIALS AND METHODS

### 2.1 Study site

Huangshan (30°01′–30°18′  N, 118°01′–118°17′  E) is a high mountain in eastern China. It is located north of Huangshan City, Anhui Province, spanning five counties, covering an area of ~1200 ha. The Huangshan Mountain area is important for China–Japan flora, and it is a region with high biodiversity in East Asia. Studies revealed that the mountains surrounded by Mt. Tianmu, Mt. Huangshan, and Mt. Huaiyu may have acted as refuges for *Quercus chenii* and *Ginkgo biloba* during the last ice age (Li, Zhang, et al., 2019; Zhao et al., 2019) The Huangshan forest dynamics plot (HS) was established in 2014, and a re‐census was performed in 2019 (Xie et al., 2022). The actual coordinates of the forestry dynamics plot are 30°8′26″ N, 116°6′38″ E, and an elevation of 400–600 m, with an annual average temperature of 7.8°C. The 10.24 ha plot was divided into 256 quadrats (20 m × 20 m), in which species diversity (species richness, Shannon–Wiener index, etc.) and topography (convexity, altitude, slope, and aspect) have been previously measured (Xie et al., 2022). A 20 m × 20 m quadrat can be decomposed into 16 5 m × 5 m and 4 10 m × 10 m quadrats, respectively. The whole plot was divided into small (5 m × 5 m), medium (10 m × 10 m and 20 m × 20 m), and large (40 m × 40 m) scales. The dominant species in the HS are *Castanopsis eyrei* and *Pinus massoniana*.

### 2.2 Measuring topographic and species diversity

The CTFS approach was used to calculate four topographic factors. In each 20 × 20 m quadrat, the mean of four vertices was used as the elevation of each quadrat, and the angles between the plane formed by any three angles of the target quadrat were used to calculate the average. The horizontal plane was taken as the slope of the quadrat, and the average deviation angle from the true north direction. The elevation of the target quadrat minus the average elevation of the surrounding eight squares was used as the convexity of the quadrat.

Four species diversity indices (species richness, the Shannon–Wiener index, Shannon–Wiener diversity, and Pielou index) were calculated using the vegan package (Oksanen et al., 2015). All indices were based on the 2019 re‐census data. The four indices were calculated as follows (Magurran, 1988):

Species richness:

(1) S=n,

where *S* is the species richness in plots of different scales, and *n* is the number of tree species with a diameter at breast height ≥1 cm.

Shannon–Wiener index:

(2) $$H=-\sum_{i=1}^{S}p_{i}\log_{x}p_{i},$$

where *p*_*i*_ is the relative abundance of species *i*, *p*_*i*_ = *N*_*i*_/*N*_0_, *N*_*i*_ is the abundance of *i* species, and *N*_0_ is the sum of the abundances of all species.

Shannon–Wiener diversity:

(3) $$H^{\prime }=e^{H},$$

where *H* is the Shannon–Wiener index of quadrats, and *H*′ is the Shannon–Wiener diversity.

Pielou index:

(4) $$J=\frac{H}{\log_{x}S},$$

where *H* is the Shannon–Wiener index, and *S* is the tree richness.

### 2.3 Measuring aboveground biomass and productivity

In this study, the methods of Gonzalez‐Akre et al. (2021) were used to measure aboveground biomass. This is a recent database of allometric equations. Differences caused by sample size, climate, and taxonomy are weighted, making the calculation of aboveground biomass more precise. Herein, the relative change in biomass in each quadrat was used to calculate the productivity of the community (Lasky et al., 2014). The calculation was performed using the following equation:

(5) $$P=\left({\text{Biomass}}_{2014}/{\text{Biomass}}_{2019}\right)/t,$$

where *P* is the productivity of the quadrat, Biomass_2014_ is the sum of alive biomass in plots of different scales in 2014, Biomass_2019_ is the sum of alive biomass in plots of different scales in 2019, and t is the time interval from 2014 to 2019.

### 2.4 Data analysis

All 5 m × 5 m and 10 m × 10 m quadrats with no recorded species or with outliers were excluded. In total, 3968 5 m × 5 m quadrats, 1021 10 m × 10 m quadrats, 256 20 m × 20 m quadrats, and 64 40 m × 40 m quadrats were included in this study. To explore the relationships between species diversity and biomass at different scales, we used a generalized linear regression model with a Gamma error structure and log/inverse link function. The Akaike Information Criterion (AIC) was used to compare the two models constructed by two different link functions. We chose a model with a smaller AIC value. Considering productivity as ratio data, we used generalized linear regression models with gamma error structure, quasi‐Poisson error structure, and negative binomial error structure through the log link function to fit the relationship between productivity and species diversity. AIC was used to evaluate how well the three models fitted, and we chose a model with a smaller AIC value. The check_overdispersion function in the ‘performance’ package was used to assess the model in which the Poisson error was overdispersed. Generalized linear regression models with a quasi‐Poisson error structure were used to reduce the probability of type I error.

Theoretical causal links between AGB, environmental drivers, and forest attributes are well understood and can form a nice causal relationship (Rodríguez‐Hernández et al., 2021). The structural equation model (SEM) was used to explore causal relationships between species diversity, topographic factors, biomass, and productivity at a scale of 20 m × 20 m. First, we built a meta‐model based on assumptions and theoretical relationships among species diversity, topography, biomass, and productivity, using the piecewiseSEM package (version: 2.2; Figure S1; Lefcheck, 2016). Secondly, *D*‐separation was used to test the fit of the model, and Fisher's *C* > 0.05 indicated a good fit. All data analysis was performed in R version 4.1.1.

## 3 RESULTS

### 3.1 Relationships between species diversity and aboveground biomass

**FIGURE 1:**
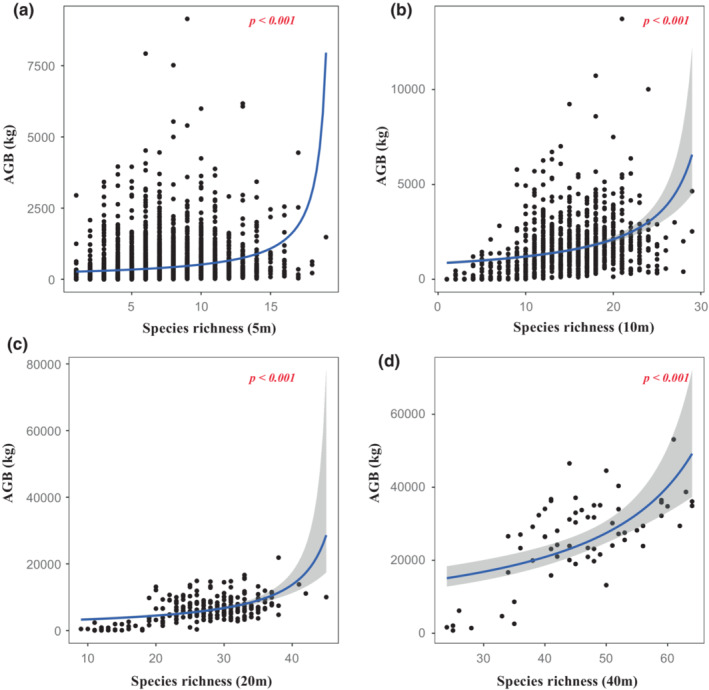
Relationships between species richness and aboveground biomass (AGB) at different scales. (a) 5 m × 5 m; (b) 10 m × 10 m; (c) 20 m × 20 m; (d) 40 m × 40 m.

**FIGURE 2:**
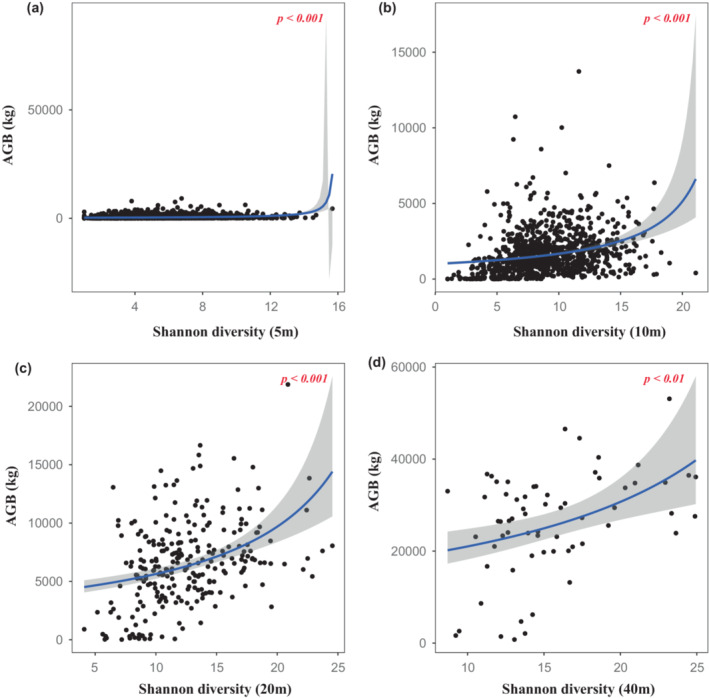
Relationships between Shannon diversity and aboveground biomass (AGB) at different scales. (a) 5 m × 5 m; (b) 10 m × 10 m; (c) 20 m × 20 m; (d) 40 m × 40 m.

**FIGURE 3:**
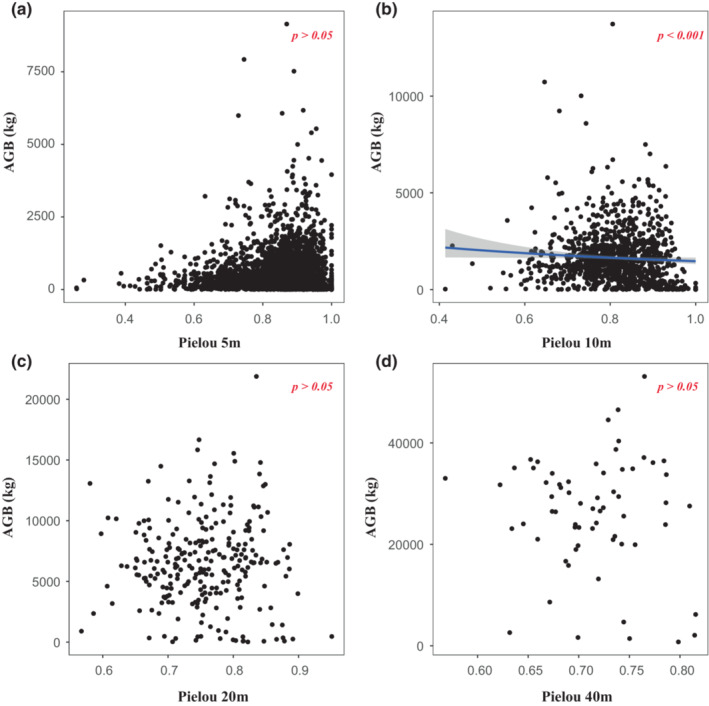
Relationships between Pielou index and aboveground biomass (AGB) at different scales. (a) 5 m × 5 m; (b) 10 m × 10 m; (c) 20 m × 20 m; (d) 40 m × 40 m.

Two alternative models were assessed, including log and inverse transformation. We compared two models and found that model with log transformation yielded lower AIC (Table 1). Based on analysis of the optimal model, we used the generalized linear regression model with gamma error structure and log link function. We found that both species richness and Shannon diversity were positively correlated with aboveground biomass (Table 2, Figures 1 and 2). However, although a positive relationship was observed between the Pielou index and aboveground biomass in 10 m × 10 m quadrats (Figure 3b), there was no linear relationship between the Pielou index and aboveground biomass at the other three scales (Figure 3a–d). The effects of species diversity on aboveground biomass decreased with the increase of spatial scale (Table 1). These suggest that scale‐dependent was significant found in relationships between species diversity and aboveground biomass.

**TABLE 1:** Analysis of the optimal model linking species diversity and aboveground biomass at different scales.

Species diversity	Scale	Inverse link			Log link		
		AIC	Coefficient	Intercept	AIC	Coefficients	Intercept
Species richness	5 m × 5 m	54,621	−0.0002	0.004	**54,526**	0.13	5.07
	10 m × 10 m	17,074	−3.6 × 10^−5^	0.0012	**17,023**	0.09	6.02
	20 m × 20 m	4929.5	−7.6 × 10^−6^	3.7 × 10^−4^	**4902.1**	0.07	6.70
	40 m × 40 m	1397	−1.1 × 10^−6^	9.4 × 10^−5^	**1389.6**	0.04	8.14
Shannon–Wiener diversity	5 m × 5 m	54,648	−2.3 × 10^−4^	3.7 × 10^−3^	**54,562**	0.17	5.10
	10 m × 10 m	17,115	−4.0 × 10^−5^	0.001	**17,023**	0.09	6.02
	20 m × 20 m	49,58.5	−7.4 × 10^−6^	2.5 × 10^−4^	**4954.7**	0.06	7.99
	40 m × 40 m	1407.5	−1.5 × 10^−6^	6.2 × 10^−5^	**1407.2**	0.04	9.47
Pielou Index	5 m × 5 m	53,077	0.3 × 10^−3^	0.002	53,077	−0.17	6.23
	10 m × 10 m	17,108	0.3 × 10^−3^	0.3 × 10^−3^	**17,023**	0.09	6.02
	20 m × 20 m	4978.2	4.4 × 10^−6^	1.5 × 10^−4^	4978.2	−0.03	8.8
	40 m × 40 m	1412.2	3.0 × 10^−5^	1.6 × 10^−5^	1412.2	−0.86	10.8

*Note*: Bold font indicates that the model was selected to fit linear regression.

**TABLE 2:** Analysis of the optimal model between species diversity and aboveground biomass at different scales.

Species diversity	Scale	Negative binomial			Quasipoisson		Gamma		
		Coefficient estimation	Intercept	*p*	Coefficient estimation	Intercept	Coefficient estimation	Intercept	*p*
Species richness	5 m × 5 m	0.008	−1.31	.36	0.007	−1.31	0.008	−1.31	.61
	10 m × 10 m	0.04	−2.07	<.001	0.04	−2.06	0.06	−2.35	**<.001**
	20 m × 20 m	0.04	−2.51	.09	0.04	−2.51	0.06	−3.14	**<.001**
	40 m × 40 m	0.02	−2.59	.45	0.02	−2.59	0.31	−3.07	**<.001**
Shannon–Wiener diversity	5 m × 5 m	0.02	−1.35	.13	0.02	−1.35	0.02	−1.35	.39
	10 m × 10 m	0.06	−1.99	<.01	0.06	−1.99	0.08	−2.11	**<.001**
	20 m × 20 m	0.03	−1.92	.32	—	—	0.04	−2.02	**.02**
	40 m × 40 m	0.02	1.92	.74	—	—	0.02	−1.95	.054
Pielou Index	5 m × 5 m	0.02	−0.35	.13	0.60	−1.75	0.56	−1.71	.27
	10 m × 10 m	0.06	−1.99	**.002**	−0.13	−1.30	−0.15	−1.28	.87
	20 m × 20 m	0.03	−1.92	.32	—	—	−0.70	−0.97	.50
	40 m × 40 m	0.21	−1.92	.74	—	—	−1.99	−1.17	**.04**

*Note*: The *p* value for the negative binomial model with gamma error and log link indicates whether the model is significant. Bold font indicates that the model was selected to fit linear regression.

### 3.2 Relationships between species diversity and productivity

**FIGURE 4:**
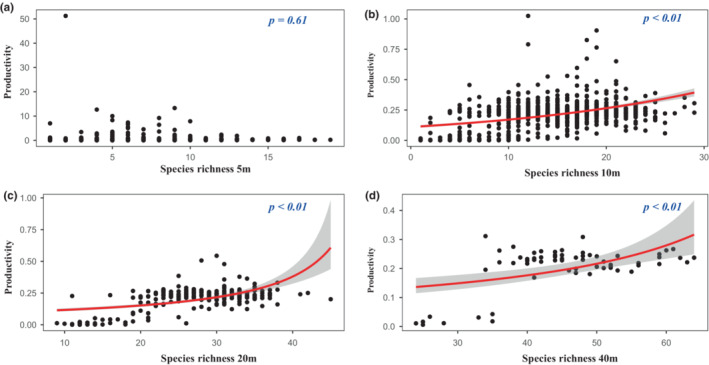
Relationships between species richness and productivity at different scales. (a) 5 m × 5 m; (b) 10 m × 10 m; (c) 20 m × 20 m; (d) 40 m × 40 m.

**FIGURE 5:**
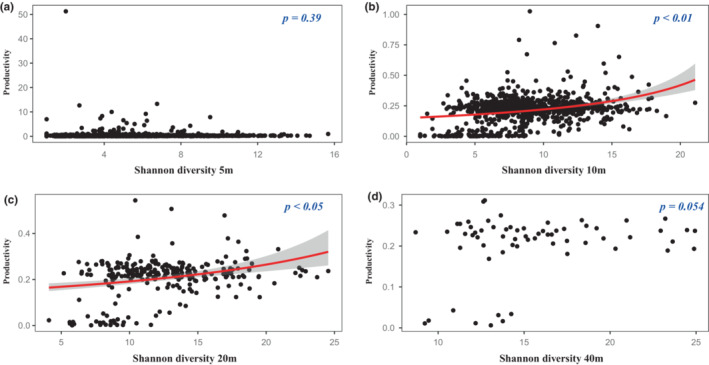
Relationships between Shannon diversity and productivity at different scales. (a) 5 m × 5 m; (b) 10 m × 10 m; (c) 20 m × 20 m; (d) 40 m × 40 m.

**FIGURE 6:**
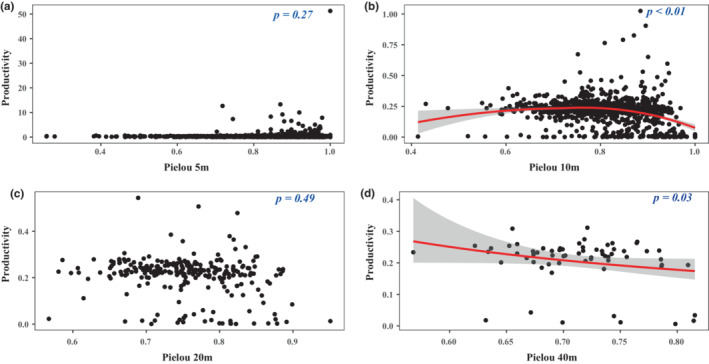
Relationships between Pielou index and productivity at different scales. (a) 5 m × 5 m; (b) 10 m × 10 m; (c) 20 m × 20 m; (d) 40 m × 40 m.

We firstly used a generalized linear model with gamma error structure and log link function to fit a curve to explore the relationship between species diversity and productivity (Figure 4). The impacts of species diversity on productivity at different scales were mostly positive, but the impacts of species diversity on productivity were not correlate with spatial scale. Figure 4 shows that a positive relationship was found between species richness and productivity, while no relationships were observed at the 5 m × 5 m scale (Figure 4a). At 20 m × 20 m and 10 m × 10 m scales, positive relationships were found, but no correlation was observed at the other two scales (Figure 5). Only a negative relationship between Pielou index and productivity was found at the 40 m × 40 m (Figure 6d). Considering that productivity is ratio data and that most values are small, we fitted the relationship between productivity and species diversity based on negative binomial regression with the log link function. It is worth noting that at the 10 m × 10 m scale, we only found that the negative binomial distribution was a good fit for the relationship between productivity and Pielou index (Figure 6b).

### 3.3 Causal relationships among topography, species diversity, biomass, and productivity

**FIGURE 7:**
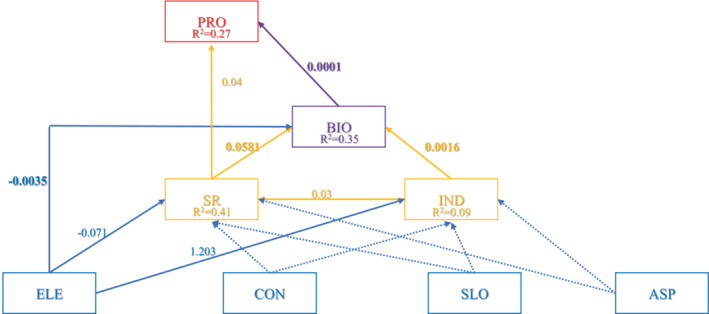
Structural equation model depicting the influence of topography factors and species diversity on productivity and biomass. Solid and dotted lines represent significant and nonsignificant relationships, respectively. ASP, aspect; BIO, biomass; CON, convexity; ELE, elevation; IND, number of individuals in 20 m × 20 m quadrats; PRO, productivity; SLO, slope; SR, species richness.

Structural equation model indicated that elevation had a direct negative effect on species richness and biomass, but a direct positive effect on the number of individuals in quadrats (Figure 7). Both species richness and the number of individuals had direct positive effects on biomass and indirect positive effects on productivity (Figure 7), while convexity, slope, and aspect had no effect on species diversity, biomass, or productivity (Figure 7). We also found that elevation had a direct negative effect on biomass, but no direct effect on productivity. The Fisher *C* value of the whole model was 10.32, indicating that the model fit well (*p* = .738).

## 4 DISCUSSION

The impacts of biodiversity on ecosystem function have been ambiguous. In particular, the mismatch between the results of control experiments and natural observation experiments in forest ecosystems has caused certain difficulties for us to understand BEF in complex ecosystems. The effects of different biotic or abiotic factors on forest community assembly and ecosystem functioning have been extensively studied, and species diversity is considered a key factor for ecosystem functioning (Huang et al., 2021). However, these still stayed on the global and regional scales and obtained a macro result and ignored the possible scale effect on BEF (Chisholm et al., 2013; Li, Bao, et al., 2019; Liang et al., 2016). For the first time, we examined the effects of environmental heterogeneity on species diversity, biomass, and productivity in subtropical evergreen broadleaved forests in China at a local scale. Overall, our results indicated a positive correlation between species diversity and ecosystem function, still ambiguous relationships were found for BEF due to scale effects. This suggested that it could not be generalized at larger scales; hence, it remains challenging to protect forest ecosystems.

### 4.1 Scale‐dependent effects of species diversity on biomass and productivity

In this study, a positive relationship was found between species richness and aboveground biomass at different sampling scales (Figure 1), which is consistent with an observational experiment in secondary forests (Wu et al., 2018). It indicates that biodiversity effect has significant impact on the carbon stock of subtropical forests. The increase of species richness is conducive to the increase of the opportunity to include species with high‐carbon storage in the community, which makes the increase of total biomass (Wu et al., 2018).

Shannon diversity, a diversity index based on abundance, can give different weights to rare species and common species to better represent the level of species diversity (Jing et al., 2020). Consistent with species richness, we also found a positive correlation between Shannon diversity and aboveground biomass (Figure 2). Some studies have reported that Shannon diversity can increase initially with scale and then stabilize thereafter and that Shannon diversity can also continually expand with spatial scale (Ye et al., 1998). However, in the present study, we found that aboveground biomass first stabilized and then increased rapidly with Shannon diversity. This shows that the ecological process affecting biomass accumulation in the plot is related to the resource utilization caused by shannon diversity. Different species occupy different niches in the same community. Plant communities with high Shannon diversity suggested that the more homogeneous individual distribution among species, the more obvious niche differentiation (Ali et al., 2016). Plants used all kinds of resources more effectively and obtained higher productivity.

Additionally, the effect of scale on species evenness and biomass was observed, and there was no correlation between the Pielou evenness index and biomass at small and large scales (Figure 3). Some studies have found that taxonomy has a significant effect on accumulation of biomass, due to differences in functional diversity and resource utilization efficiency between species (Li, Bao, et al., 2019; Lin et al., 2012). In HS, *C. eyrei* is a major component of the community that competes with other species for resources at a small scale (Ding et al., 2016; Xie et al., 2022). *C. eyrei* was the species with the highest importance value in most of the quadrats (Table S1). Therefore, due to higher spatial utilization, *C. eyrei* has an impact on the resource utilization of other species, through more efficient use of light for carbon storage. We can infer that the relationship between Pielou index and ecosystem function is affected by the accumulation of higher biomass in areas where *C. eyrei* are most suited to the environmental conditions. This suggests that species abundance and size play more important roles in species diversity than species uniform distribution. Therefore, when analyzing regional species diversity at the local scale, the scale effect of the sampling area should be considered, and an appropriate species diversity index should be selected.

A positive relationship between species diversity and productivity was observed at the medium scale (Figures 4, 5, 6). These findings are consistent with previous studies on biodiversity–productivity relationships in global forests, increasing total productivity in forest ecology system (Liang et al., 2016). Species might have different resource utilization, the higher the species diversity, the stronger the mutual promotion among species in the community (Che & Jin, 2019). These suggest that the niche complementarity hypothesis plays an important role in the relationship between species diversity and productivity. By comparing the importance values from 2014 to 2019, it was found that there was no major succession of species in the plot, which indicates that the Huangshan community assembly was stable, and the utilization of species resources and niche differentiation were reasonable. A subtropical natural forest ecosystem in late successional stages may eliminate some less productive species and retain some more productive species. Furthermore, complementarity and facilitation will be of high value under such a scenario, in which case the influence of dominant species on community ecosystem function becomes more important (Carreño‐Rocabado et al., 2012). In addition, one study demonstrated that niche complementarity effects mainly occur at small scales such as 20 m × 20 m subplots (Chisholm et al., 2013).

In general, species diversity is divided into species numbers within the community based on importance value, coverage, and species richness and species evenness (He et al., 2003). When the species richness and species evenness of the community are higher, the species diversity of the community is higher (Purvis & Hector, 2000). In such cases, due to competition for resources and succession, some species with higher‐carbon storage capacity can become key factors determining the total biomass of the community. On the other hand, abundance‐based Shannon diversity can better represent the true level of species diversity by assigning different weights to rare and common species (Jing et al., 2020). In fact, it is not the first time that the effects of evenness on ecosystem functioning have been reported. The results of a control experiment showed that the total biomass and aboveground biomass of a community increased linearly with increasing evenness and did not change with community types (Wilsey & Potvin, 2000). Therefore, we can conclude that at a certain scale, changes in species composition alter forest carbon storage by altering canopy structure and thus photosynthesis (Catovsky & Bazzaz, 2000). In the present work, we found a positive correlation between species evenness and productivity. However, no correlation was observed between species diversity and productivity (Figures 4a, 5a, and 6a). This indicates scale‐dependent effects on the relationships between productivity and biodiversity. This indeterminate relationship between species diversity and productivity is not the first to be discovered. In tropical forest on Barro Colorado Island, Panama, the effects of species richness on aboveground biomass were increased with increasing sampling area (Tan et al., 2017). In a grassland ecosystem, Adler et al. (2011) found that species diversity was not a good predictor of community productivity at different sampling scales. A possible explanation is that the available resources not only affect the maximum biomass that can be achieved by species in the area but also cause biomass to stop increasing (Zhu et al., 2021). On the other hand, scale can also lead to resource competition among species, thereby affecting the number of species. In 20 m × 20 m and 40 m × 40 m quadrats, we observed a negative relationship and no relationship between species evenness and biomass/productivity, respectively (Tables 1 and 2). This indicates that the number of species increased with increasing sampling area size, but the biomass and productivity of species did not increase. One possible explanation is that environmental heterogeneity increases as the sampling area increases, which leads to inconsistent effects of habitat on species diversity versus biomass and productivity against niche complementarity and sampling effects (Ma et al., 2010; Prado‐Junior et al., 2016). Overall, species diversity increases ecosystem function at local scales.

We mostly observed monotonic increases between species diversity and biomass or productivity. A possible explanation is that the number of species at the local scale is small, the competition for resources is low, and the functional complementarity of species is strong; hence, we only observed that ecosystem functioning increases with increasing species diversity at the local scale (Turner & Tjørve, 2005). Che and Jin (2019) reported similar findings in that phylogenetic diversity had a stronger impact on productivity than species diversity at small scales, while the opposite was true at large scales. In a large‐scale subtropical forest experiment, species richness was negatively correlated with stand‐volume changes when species richness was high and then positively correlated with stand‐volume changes when species richness was low (Huang et al., 2018). This indicates that more species can be accommodated at large scales and that greater spatial heterogeneity can provide more ecological niches, resulting in less competition for resources among species. A similar result was also observed in a region‐scale observational experiment. At 10 m × 10 m resolution, species diversity has no correlation with biomass. However, at 20 m × 30 m resolution, species diversity is positively correlated with biomass (Ouyang et al., 2016). In summary, there may be a positive correlation between species diversity and biomass/productivity in large‐scale sampling areas. Furthermore, when analyzing BEF at the local scale, the scale effect of the sampling area should be considered, and an appropriate species diversity index should be selected.

### 4.2 Effects of species richness and topography on ecosystem functions

In our SEM analysis, the number of individuals directly affected biomass and indirectly affected productivity through biomass, and both effects were positively correlated. One possible explanation is that both species diversity and ecosystem functioning are affected by abiotic factors such as resource allocation (Ma et al., 2010). Environmental filtration preserves some species with higher productivity but decreases species diversity and resource competition (Grime, 1998; Laliberte et al., 2014). Therefore, we inferred that environmental heterogeneity has a positive effect on productivity through species richness at the local scale. However, we found that both the direct effect of species diversity on productivity and the indirect effect and total effect combined; hence, their effect on productivity appeared to be minimal (Figure 7). A growing body of research suggests a stronger relationship between functional diversity and productivity, compared with species diversity (Bongers et al., 2020; Mokany et al., 2008; Wen & Jin, 2019). Thus, future work should examine the key drivers of forest productivity across multiple biodiversity perspectives.

In addition to species richness and the number of individuals, we found that topographic factors had direct and indirect effects on biomass and productivity (Figure 7). Specifically, elevation may be a key factor affecting the biomass and productivity of evergreen broadleaved forests in HS. We revealed a negative effect of elevation on biomass and species richness (Figure 7). It is widely accepted that alpha‐diversity decreases with increasing elevation (Liu et al., 2012), and a decrease in the number of species will inevitably lead to a decrease in biomass (Vile et al., 2006). Therefore, elevation may be an important factor affecting biomass and species diversity in HS. The positive and negative effects of elevation on species richness and the number of individuals indicate the complexity of the effects of topographic factors. Changes in topographic factors would inevitably alter the influence of light, water, and nutrients on plant community assembly and indirectly affect aboveground biomass and productivity (Wen & Jin, 2019; Zhu et al., 2021). Therefore, further research is needed to explore the impacts of environmental factors such as topography on aboveground biomass and productivity.

Stands with high species diversity often have more complex stand structures and edge effects, and such stands can better utilize sunlight and soil fertility, thereby changing the microclimate in the forest and improving the growth environment in forest areas (Zhu et al., 2021). Furthermore, this can increase the nutrient content of the soil, improve the speed of nutrient flow and circulation, and continuously improve soil fertility, resulting in more new tree species in a given plot, decreased tree mortality, and allowing existing trees to obtain higher biomass (Wu et al., 2018; Zhu et al., 2021). Future studies should explore the effects of soil nutrients, light, and other environmental factors on biomass, productivity, and species community structure.

## 5 CONCLUSION

We revealed a positive relationship between BEF based on analysis of species diversity and productivity/biomass. However, a mismatch between productivity and biodiversity was found at small and large scales, indicating that the effects of scale are dependent on BEF. Meanwhile, scale‐independent effects had less influence on biomass than productivity. Furthermore, we observed significant effects of biotic and abiotic factors on biodiversity and ecosystem functioning at the medium scale. Through measuring forest biomass, we can explore the growth status of trees in the recent past, as well as current and future growth trends, and provide effective and reliable data for forest survey planning, afforestation design, and scientific management of the ecological environment.

## AUTHOR CONTRIBUTIONS

**Lei Xie:** Conceptualization (lead); formal analysis (lead); methodology (lead); software (lead); writing – original draft (lead). **Hao Chen:** Data curation (lead); resources (lead). **Lai Wei:** Conceptualization (equal); data curation (lead); resources (equal); writing – original draft (equal). **Shuifei Chen:** Funding acquisition (equal); project administration (equal); resources (equal). **Lu Wang:** Investigation (lead); resources (equal). **Baokun Xu:** Investigation (equal); resources (equal). **Xiangui Yi:** Investigation (equal); resources (equal). **Xianrong Wang:** Investigation (equal); resources (equal); supervision (equal). **Hui Ding:** Funding acquisition (equal); investigation (equal); project administration (equal); resources (equal). **Yanming Fang:** Funding acquisition (lead); investigation (equal); methodology (lead); project administration (equal); writing – original draft (equal); writing – review and editing (lead).

## FUNDING INFORMATION

This research was funded by the Special Foundation for National Science and Technology Basic Re‐sources Investigation of China (grant number 2019FY202300), the Basic Scientific Research Funds Programs in the National Public Welfare Research Institutes of China (grant number GYZX200203, 210503), and the Major Biodiversity Conservation Project of the Ministry of Ecology and Environment (grant number ZDGC2019‐007‐04).

## CONFLICT OF INTEREST STATEMENT

The authors declare that they have no known competing financial interests or personal relationships that could have appeared to influence the work reported in this paper.

## Supporting information


Appendix S1

## Data Availability

Data analyzed in this study are publicly available at Figshare (https://figshare.com/articles/dataset/HS_PRO‐SD/19129496).

## References

[ece39786-bib-0001] Adler, P. B. , Seabloom, E. W. , Borer, E. T. , Hillebrand, H. , Hautier, Y. , Hector, A. , Harpole, W. S. , O'Halloran, L. R. , Grace, J. B. , & Anderson, T. M. (2011). Productivity is a poor predictor of plant species richness. Science, 333, 1750–1753. 21940895 10.1126/science.1204498

[ece39786-bib-0002] Ali, A. , Yan, E. R. , Chen, H. , Chang, S. X. , Zhao, Y. T. , & Yang, X. D. (2016). Stand structural diversity rather than species diversity enhances aboveground carbon storage in secondary subtropical forests in eastern China. Biogeosciences, 13(16), 4627–4635.

[ece39786-bib-0003] Bond, E. M. , & Chase, J. M. (2002). Biodiversity and ecosystem functioning at local and regional spatial scales. Ecology Letters, 5, 467–470.

[ece39786-bib-0004] Bongers, F. J. , Schmid, B. , Durka, W. , Li, S. , Bruelheide, H. , Hahn, C. Z. , Yan, H. R. , Ma, K. , & Liu, X. (2020). Genetic richness affects trait variation but not community productivity in a tree diversity experiment. The New phytologist, 227, 744–756. 32242938 10.1111/nph.16567

[ece39786-bib-0005] Carreño‐Rocabado, G. , Peña‐Claros, M. , Bongers, F. , Alarcón, A. , Licona, J. C. , Poorter, L. , & Vesk, P. (2012). Effects of disturbance intensity on species and functional diversity in a tropical forest. Journal of Ecology, 100, 1453–1463.

[ece39786-bib-0006] Catovsky, S. , & Bazzaz, F. A. (2000). Contributions of coniferous and broad‐leaved species to temperate forest carbon uptake: A bottom‐up approach. Canadian Journal of Forest Research, 30, 100–111.

[ece39786-bib-0007] Cavanaugh, K. C. , Gosnell, J. S. , Davis, S. L. , Ahumada, J. , Boundja, P. , Clark, D. B. , Mugerwa, B. , Jansen, P. A. , O'Brien, T. G. , Rovero, F. , Sheil, D. , Vasquez, R. , & Andelman, S. (2014). Biodiversity and aboveground carbon storage. Global Ecology and Biogeography, 23, 563–573.

[ece39786-bib-0008] Chase, J. M. , & Leibold, M. A. (2002). Spatial scale dictates the productivity–biodiversity relationship. Nature, 416, 427–430. 11919631 10.1038/416427a

[ece39786-bib-0009] Chave, J. (2013). The problem of pattern and scale in ecology: What have we learned in 20 years? Ecology Letters, 16, 4–16. 23351093 10.1111/ele.12048

[ece39786-bib-0010] Che, Y. , & Jin, G. Z. (2019). Effects of species diversity and phylogenetic diversity on productivity of a mixed broadleaved‐Korean pine forest. Chinese Journal of Applied Ecology, 30, 2241–2248. 31418226 10.13287/j.1001-9332.201907.010

[ece39786-bib-0011] Chisholm, R. A. , Muller‐Landau, H. C. , Abdul Rahman, K. , Bebber, D. P. , Bin, Y. , Bohlman, S. A. , Bourg, N. A. , Brinks, J. , Bunyavejchewin, S. , Butt, N. , Cao, H. , Cao, M. , Cárdenas, D. , Chang, L.‐W. , Chiang, J.‐M. , Chuyong, G. , Condit, R. , Dattaraja, H. S. , Davies, S. , … Zimmerman, J. K. (2013). Scale‐dependent relationships between tree species richness and ecosystem function in forests. Journal of Ecology, 101, 1214–1224.

[ece39786-bib-0012] Ding, H. , Fang, Y. M. , Yang, X. H. , Yuan, F. Y. , He, L. H. , Yao, J. , Wu, J. , Chi, B. , Li, Y. , Chen, S. F. , Chen, T. , & Xu, H. (2016). Community characteristics of a subtropical evergreen broad‐leaved forest in Huangshan, Anhui Province, East China. Biodiversity Science, 24, 875–887.

[ece39786-bib-0013] Gaston, K. J. (2000). Global patterns in biodiversity. Nature, 405, 220–227. 10821282 10.1038/35012228

[ece39786-bib-0014] Gillman, L. N. , & Wright, S. D. (2006). The influence of productivity on the species richness of plants: A critical assessment. Ecology, 87, 1234–1243. 16761602 10.1890/0012-9658(2006)87[1234:tiopot]2.0.co;2

[ece39786-bib-0015] Gonzalez‐Akre, E. , Piponiot, C. , Lepore, M. , Herrmann, V. , Lutz, J. A. , Baltzer, J. L. , Dick, C. W. , Gilbert, G. S. , He, F. , & Heym, M. (2021). allodb: An R package for biomass estimation at globally distributed extratropical forest plots. Methods in Ecology and Evolution, 13, 330–338.

[ece39786-bib-0016] Grime, J. (1998). Benefits of plant diversity to ecosystems: Immediate, filter and founder effects. Journal of Ecology, 86, 902–910.

[ece39786-bib-0017] He, J. S. , Fang, J. Y. , Ma, K. P. , & Huang, J. H. (2003). Biodiversity and ecosystem productivity: Why is there a discrepancy in the relationship between experimental and natural ecosystems? Chinese Journal of Plant Ecology, 27, 835–843.

[ece39786-bib-0018] Huang, X. , Lang, X. , Li, S. , Liu, W. , & Su, J. (2021). Indicator selection and driving factors of ecosystem multifunctionality: Research status and perspectives. Biodiversity Science, 29, 1673–1686.

[ece39786-bib-0019] Huang, Y. Y. , Chen, Y. X. , Castro‐Izaguirre, N. , Baruffol, M. , Brezzi, M. , Lang, A. , Li, Y. , Härdtle, W. , von Oheimb, G. , Yang, X. , Liu, X. , Pei, K. , Both, S. , Yang, B. , Eichenberg, D. , Assmann, T. , Bauhus, J. , Behrens, T. , Buscot, F. , … Schmid, B. (2018). Impacts of species richness on productivity in a large‐scale subtropical forest experiment. Science, 362(6410), 80–83. 30287660 10.1126/science.aat6405

[ece39786-bib-0020] Jing, X. , Prager, C. M. , Classen, A. T. , Maestre, F. T. , He, J. , & Sanders, N. J. (2020). Variation in the methods leads to variation in the interpretation of biodiversity‐ecosystem multifunctionality relationships. Journal of Plant Ecology, 13, 431–441.

[ece39786-bib-0021] Kurz, W. A. , & Apps, M. J. (1993). Contribution of northern forests to the global C cycle: Canada as a case Study. Water Air & Soil Pollution, 70(1), 163–176.

[ece39786-bib-0022] Laliberte, E. , Zemunik, G. , & Turner, B. L. (2014). Environmental filtering explains variation in plant diversity along resource gradients. Science, 345, 1602–1605. 25258078 10.1126/science.1256330

[ece39786-bib-0023] Lasky, J. R. , Uriarte, M. , Boukili, V. K. , Erickson, D. L. , John, K. W. , & Chazdon, R. L. (2014). The relationship between tree biodiversity and biomass dynamics changes with tropical forest succession. Ecology Letters, 17, 1158–1167. 24986005 10.1111/ele.12322

[ece39786-bib-0024] Lawton, J. H. (1994). What do species do in ecosystems? Oikos, 71, 367–374.

[ece39786-bib-0025] Lefcheck, J. S. (2016). piecewiseSEM: Piecewise structural equation modelling in R for ecology, evolution, and systematics. Methods in Ecology and Evolution, 7, 573–579.

[ece39786-bib-0026] Li, Y. , Bao, W. K. , Bongers, F. , Chen, B. , Chen, G. K. , Guo, K. , Jiang, M. X. , Lai, J. S. , Lin, D. M. , Liu, C. J. , Liu, X. J. , Liu, Y. , Mi, X. C. , Tian, X. J. , Wang, X. H. , Xu, W. B. , Yan, J. H. , Yang, B. , Zheng, Y. R. , & Ma, K. P. (2019). Drivers of tree carbon storage in subtropical forests. Science of the Total Environment, 654, 684–693. 30448659 10.1016/j.scitotenv.2018.11.024

[ece39786-bib-0027] Li, Y. , Zhang, X. W. , & Fang, Y. M. (2019). Landscape features and climatic forces shape the genetic structure and evolutionary history of an oak species (*Quercus chenii*) in East China. Frontiers in Plant Science, 10, 1060. 31552065 10.3389/fpls.2019.01060 PMC6734190

[ece39786-bib-0028] Liang, J. , Crowther, T. W. , Picard, N. , Wiser, S. , Zhou, M. , Alberti, G. , Schulze, E. D. , McGuire, A. D. , Bozzato, F. , & Pretzsch, H. (2016). Positive biodiversity‐productivity relationship predominant in global forests. Science, 354, aaf8957. 27738143 10.1126/science.aaf8957

[ece39786-bib-0029] Lin, D. , Lai, J. , Muller‐Landau, H. C. , Mi, X. , & Ma, K. (2012). Topographic variation in aboveground biomass in a subtropical evergreen broad‐leaved forest in China. PLoS One, 7, e48244. 23118961 10.1371/journal.pone.0048244 PMC3484055

[ece39786-bib-0030] Liu, H. , Xue, D. , & Sang, W. (2012). Effect of topographic factors on the relationship between species richness and aboveground biomass in a warm temperate forest. Ecology and Environmental Sciences, 21, 1403–1407.

[ece39786-bib-0031] Lv, T. , Wang, N. , Xie, L. , Chen, S. , Zhao, R. , Feng, Y. , Li, Y. , Ding, H. , & Fang, Y. (2022). Environmental heterogeneity affecting community assembly patterns and phylogenetic diversity of three forest communities at Mt. Huangshan, China. Forests, 13, 133.

[ece39786-bib-0032] Ma, W. , He, J. S. , Yang, Y. , Wang, X. , Liang, C. , Anwar, M. , Zeng, H. , Fang, J. , & Schmid, B. (2010). Environmental factors covary with plant diversity–productivity relationships among Chinese grassland sites. Global Ecology and Biogeography, 19, 233–243.

[ece39786-bib-0033] Magurran, A. E. (1988). Ecological diversity and its measurement. Princeton University Press.

[ece39786-bib-0034] Mittelbach, G. G. , Steiner, C. F. , Scheiner, S. M. , Gross, K. L. , Reynolds, H. L. , Waide, R. B. , Willig, M. R. , Dodson, S. I. , & Gough, L. (2001). What is the observed relationship between species richness and productivity? Ecology, 82, 2381–2396.

[ece39786-bib-0035] Mokany, K. , Ash, J. , & Roxburgh, S. (2008). Functional identity is more important than diversity in influencing ecosystem processes in a temperate native grassland. Journal of Ecology, 96, 884–893.

[ece39786-bib-0036] Oksanen, J. , Blanchet, F. G. , Friendly, M. , Kindt, R. , Legendre, P. , McGlinn, D. , Minchin, P. R. , O'Hara, R. B. , Simpson, G. L. , & Solymos, P. (2015). Vegan: Community ecology package. 2019 . R package version, 2(10). https://cran.r‐project.org/web/packages/vegan/index.html

[ece39786-bib-0037] Olson, J. S. , Watts, J. A. , & Allison, L. J. (1983). Carbon in live vegetation of major world ecosystems. Environmental Sciences Division, Oak Ridge National Laboratory.

[ece39786-bib-0038] Oommen, M. A. , & Shanker, K. (2005). Elevational species richness patterns emerge from multiple local mechanisms in Himalayan woody plants. Ecology, 86, 3039–3047.

[ece39786-bib-0039] Ouyang, S. , Xiang, W. , Wang, X. , Zeng, Y. , Lei, P. , & Deng, X. (2016). Significant effects of biodiversity on forest biomass during the succession of subtropical forest in South China. Forest Ecology and Management, 372, 291–302.

[ece39786-bib-0040] Poorter, L. , van der Sande, M. T. , Thompson, J. , Arets, E. J. M. M. , Alarcón, A. , Álvarez‐Sánchez, J. , Ascarrunz, N. , Balvanera, P. , Barajas‐Guzmán, G. , Boit, A. , Bongers, F. , Carvalho, F. A. , Casanoves, F. , Cornejo‐Tenorio, G. , Costa, F. R. C. , de Castilho, C. V. , Duivenvoorden, J. F. , Dutrieux, L. P. , Enquist, B. J. , … Peña‐Claros, M. (2015). Carbon storage in tropical forests. Global Ecology and Biogeography, 24, 1314–1328.

[ece39786-bib-0041] Prado‐Junior, J. A. , Schiavini, I. , Vale, V. S. , Arantes, C. S. , van der Sande, M. T. , Lohbeck, M. , & Poorter, L. (2016). Conservative species drive biomass productivity in tropical dry forests. Journal of Ecology, 104, 817–827.

[ece39786-bib-0042] Purvis, A. , & Hector, A. (2000). Getting the measure of biodiversity. Nature, 405, 212–219. 10821281 10.1038/35012221

[ece39786-bib-0043] Rodríguez‐Hernández, D. I. , Deane, D. C. , Wang, W. , Chen, Y. , Li, B. , Luo, W. , & Chu, C. (2021). Direct effects of selection on aboveground biomass contrast with indirect structure‐mediated effects of complementarity in a subtropical forest. Oecologia, 196, 249–261. 33870455 10.1007/s00442-021-04915-w

[ece39786-bib-0044] Tan, S. , Wang, R. , Gong, X. , Cai, J. , & Shen, G. (2017). Scale dependent effects of species diversity and structural diversity on aboveground biomass in a tropical forest on Barro Colorado Island, Panama. Biodiversity Science, 25, 1054–1064.

[ece39786-bib-0045] Tilman, D. (1997). Distinguishing between the effects of species diversity and species composition. Oikos, 80, 185.

[ece39786-bib-0046] Tilman, D. , Lehman, C. L. , & Thomson, K. T. (1997). Plant diversity and ecosystem productivity: Theoretical considerations. Proceedings of the National Academy of Sciences of the United States of America, 94, 1857–1861. 11038606 10.1073/pnas.94.5.1857 PMC20007

[ece39786-bib-0047] Turner, W. R. , & Tjørve, E. (2005). Scale‐dependence in species‐area relationships. Ecography, 28, 721–730.

[ece39786-bib-0048] Vile, D. , Shipley, B. , & Garnier, E. (2006). Ecosystem productivity can be predicted from potential relative growth rate and species abundance. Ecology Letters, 9, 1061–1067. 16925655 10.1111/j.1461-0248.2006.00958.x

[ece39786-bib-0049] Waide, R. , Willig, M. , Steiner, C. , Mittelbach, G. , Gough, L. , Dodson, S. , Juday, G. , & Parmenter, R. (1999). The relationship between productivity and species richness. Annual Review of Ecology and Systematics, 30, 257–300.

[ece39786-bib-0050] Waring, R. H. , & Running, S. W. (2010). Forest ecosystems: Analysis at multiple scales. Elsevier.

[ece39786-bib-0051] Weiher, E. (1999). The combined effects of scale and productivity on species richness. Journal of Ecology, 87, 1005–1011.

[ece39786-bib-0052] Wen, C. , & Jin, G. (2019). Effects of functional diversity on productivity in a typical mixed broadleaved‐Korean pine forest. Chinese Journal of Plant Ecology, 43, 94–106.

[ece39786-bib-0053] Wilsey, B. , & Potvin, C. (2000). Biodiversity and ecosystem functioning: Importance of species evenness in an old field. Ecology, 81, 887–892.

[ece39786-bib-0054] Wu, C. , Han, W. , Jiang, B. , Liu, B. , Yuan, W. , Shen, A. , Huang, Y. , & Zhu, J. (2018). Relationships between species richness and biomass/productivity depend on environmental factors in secondary forests of Dinghai, Zhejiang Province. Biodiversity Science, 26, 545.

[ece39786-bib-0055] Xie, L. , Chen, S. , Feng, Y. , Li, Y. , Wang, L. , He, L. , Huang, L. , Wu, J. , Guo, K. , Ding, H. , & Fang, Y. (2022). Mismatch between specific and genetic diversity in an evergreen broadleaf forest in Southeast China: A study case of 10.24 ha forest dynamics plot of Huangshan. Frontiers in Plant Science, 12, 706006. 35173745 10.3389/fpls.2021.706006 PMC8841795

[ece39786-bib-0056] Ye, W. , Ma, K. , Ma, K. , Sang, W. , & Gao, X. (1998). Studies on plant community diversity in Donglinshan mountain, Beijing, China IX. The influence of scale on α diversity. Acta Ecologica Sinica, 18, 10–14.

[ece39786-bib-0057] Zhang, Q. , & Zhang, D. (2003). Biodiversity and ecosystem functioning: Recent advances and trends. Biodiversity Science, 11, 351–363.

[ece39786-bib-0058] Zhao, Y. P. , Fan, G. Y. , Yin, P. P. , Sun, S. , Li, N. , Hong, X. N. , Hu, G. , Zhang, H. , Zhang, F. M. , Han, J. D. , Hao, Y. J. , Xu, Q. W. , Yang, X. W. , Xia, W. J. , Chen, W. B. , Lin, H. Y. , Zhang, R. , Chen, J. , Zheng, X. M. , … Ge, S. (2019). Resequencing 545 ginkgo genomes across the world reveals the evolutionary history of the living fossil. Nature Communications, 10, 4201. 10.1038/s41467-019-12133-5 PMC6744486 31519986

[ece39786-bib-0059] Zhu, J. , Wu, A. , Zou, S. , Xiong, X. , Liu, S. , Chu, G. , Zhang, Q. , Liu, J. , Tang, X. , Yan, J. , Zhang, D. , & Zhou, G. (2021). Relationships between tree diversity and biomass/productivity and their influence factors in a lower subtropical evergreen broad‐leaved forest. Biodiversity Science, 29, 1435–1446.

