# Supplementary material for: Scale‐dependent effects of species diversity on aboveground biomass and productivity in a subtropical broadleaved forest on Mt. Huangshan
Source: Ecol Evol. 2023 Feb 1;13(2):e9786. doi: 10.1002/ece3.9786 (PMC9891959; doi:10.1002/ece3.9786)
Supplement: Supplementary file 1 — Appendix S1 [file ECE3-13-e9786-s001.docx]

Supplementary Information for

**Scale dependent effects of species diversity on aboveground biomass and productivity in a subtropical broad-leaved forest on Mt. Huangshan**

**This file includes:**

Figure S1

Table S1

**
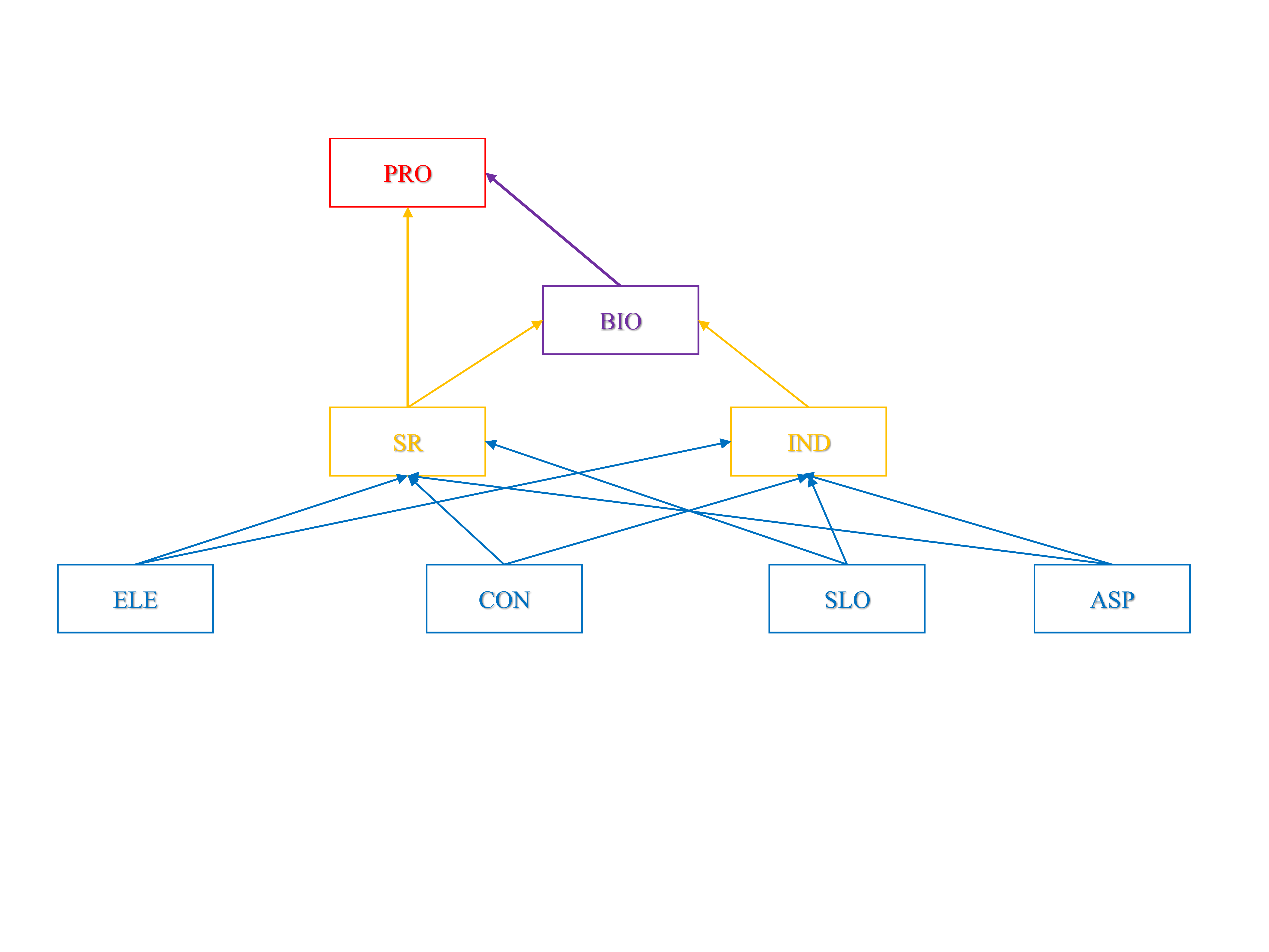
**

**Figure. S1** Meta structural equation modelling depicting the influence of topography factors and species diversity on productivity and biomass. Solid line and dotted line represent significant and no significant relationships, respectively. PRO: productivity; BIO: Biomass; SR: species richness; IND: Number of individuals in 20m × 20m subplots; ELE: elevation; CON: convexity; SLO: slope; ASP: aspect.

Table S1 Top 10 species with importance values in Huangshan Forest dynamics plot

| Species | 2019 Importance values (%) | 2014 Importance values (%) |
| --- | --- | --- |
| *Castanopsis eyrei* | 23.97 | 26.25 |
| *Eurya nitida* | 7.05 | 7.63 |
| *Rhododendron ovatum* | 6.38 | 7.60 |
| *Pinus massoniana* | 5.13 | 6.29 |
| *Loropetalum chinense* | 3.72 | 4.83 |
| *Cyclobalanopsis glauca* | 3.68 | 4.11 |
| *Vaccinium mandarinorum* | 2.84 | \ |
| *Quercus serrata* var. *brevipetiolata* | 2.41 | 2.46 |
| *Symplocos anomala* | 2.36 | 2.55 |
| *Rhododendron mariesii* | 2.23 | 2.38 |
